# A study protocol for Project I-Test: a cluster randomized controlled trial of a practice coaching intervention to increase HIV testing in substance use treatment programs

**DOI:** 10.1186/s13063-023-07602-8

**Published:** 2023-09-26

**Authors:** Jemima A. Frimpong, Carrigan L. Parish, Daniel J. Feaster, Lauren K. Gooden, Mindy C. Nelson, Tim Matheson, Karolynn Siegel, Louise Haynes, Benjamin P. Linas, Sabrina A. Assoumou, Susan Tross, Tiffany Kyle, Terri K. Liguori, Oliene Toussaint, Debra Annane, Lisa R. Metsch

**Affiliations:** 1 New York University Abu Dhabi, Saadiyat Island, PO BOX 129188, Abu Dhabi, UAE; 2 Department of Sociomedical Sciences Miami Research Center, Columbia University, 1120 NW 14 Street Room 1030, Miami, FL 33136 USA; 3 Department of Public Health Sciences, University of Miami Miller School of Medicine, 1120 NW 14Th Street, Room 1059, Miami, FL 33136 USA; 4 San Francisco Dept of Public Health (SFDPH), 25 Van Ness Avenue; Suite 500, San Francisco, CA 94102 USA; 5 Department of Sociomedical Sciences, Columbia University, 722 West 168 Street, NY, NY 10032 USA; 6 Medical University of South Carolina, 67 President Street, Charleston, SC 29425 USA; 7 Boston Medical Center, Crosstown Building, 801 Massachusetts Ave Office 2007, Boston, MA 02118 USA; 8 HIV Center For Clinical and Behavioral Studies, NYS Psychiatric Institute, Columbia University Irving Medical Center, 1051 Riverside Drive, New York, N.Y. 10032 USA; 9 Health Foundation of South Florida, 2 South Biscayne Blvd., Suite 1710, Miami, FL 33131 USA; 10 Department of Sociomedical Sciences and Columbia School of General Studies, Columbia University, 2970 Broadway, 612 Lewisohn Hall, New York, NY 10026 USA

**Keywords:** HIV, Hepatitis C virus, Practice coaching, Cluster randomized controlled trial, Organizational change, Opioid treatment program, Substance use disorder treatment

## Abstract

**Background:**

People with substance use disorders are vulnerable to acquiring HIV. Testing is fundamental to diagnosis, treatment, and prevention; however, in the past decade, there has been a decline in the number of substance use disorder (SUD) treatment programs offering on-site HIV testing. Fewer than half of SUDs in the USA offer on-site HIV testing. In addition, nearly a quarter of newly diagnosed cases have AIDS at the time of diagnosis. Lack of testing is one of the main reasons that annual HIV incidences have remained constant over time. Integration of HIV testing with testing for HCV, an infection prevalent among persons vulnerable to HIV infection, and in settings where they receive health services, including opioid treatment programs (OTPs), is of great public health importance.

**Methods/design:**

In this 3-arm cluster-RCT of opioid use disorders treatment programs, we test the effect of two evidence-based “practice coaching” (PC) interventions on the provision and sustained implementation of on-site HIV testing, on-site HIV/HCV testing, and linkage to care. Using the National Survey of Substance Abuse Treatment Services data available from SAMHSA, 51 sites are randomly assigned to one of the three conditions: practice coach facilitated structured conversations around implementing change, with provision of resources and documents to support the implementation of (1) HIV testing only, or (2) HIV/HCV testing, and (3) a control condition that provides a package with information only. We collect quantitative (e.g., HIV and HCV testing at 6-month-long intervals) and qualitative site data near the time of randomization, and again approximately 7–12 months after randomization.

**Discussion:**

Innovative and comprehensive approaches that facilitate and promote the adoption and sustainability of HIV and HCV testing in opioid treatment programs are important for addressing and reducing HIV and HCV infection rates. This study is one of the first to test organizational approaches (practice coaching) to increase HIV and HIV/HCV testing and linkage to care among individuals receiving treatment for opioid use disorder. The study may provide valuable insight and knowledge on the multiple levels of intervention that, if integrated, may better position OTPs to improve and sustain testing practices and improve population health.

**Trial registration:**

ClinicalTrials.gov NCT03135886. Registered on 2 May 2017.

**Supplementary Information:**

The online version contains supplementary material available at 10.1186/s13063-023-07602-8.

## Background

In its ongoing recognition of HIV testing as a fundamental component of HIV treatment and prevention, the latest 2022 National HIV/AIDS Strategy (NHAS) continues to encourage the expansion of HIV testing to nonclinical and nontraditional settings throughout the United States (U.S.), emphasizing the public health significance of all people with HIV (PWH) knowing their status [1]. Despite recommendations from the US Preventive Services Task Force (USPSTF) that all adolescents and adults be screened for HIV in health care settings [2], less than half (43%) of U.S. adults have ever been tested for HIV [3]. In addition, of the estimated 1.2 million PWH in the U.S., approximately 13% are unaware of their HIV status [4]; individuals unaware of their infection status are estimated to contribute to over one-third (35%) of new HIV transmissions [5, 6].

Lack of testing is considered one of the main reasons that annual HIV incidence in the U.S. has remained steady at more than 30,000 cases over the last decade [7]. The COVID pandemic exacerbated already suboptimal HIV testing efforts and led to a massive hindrance of HIV testing efforts. Over the first 1-year period of the pandemic alone (2019–2020), the Centers for Disease Control and Prevention (CDC) reported a significantly sharp decrease in testing in both healthcare (43%) and non-healthcare settings (50%) [8]. The CDC and World Health Organization (WHO) have continued to call for expanding HIV testing in settings where persons vulnerable to HIV infection receive health services, including opioid treatment programs (OTP). In addition, the 2022 NHAS called for targeted HIV efforts and resources that specifically prioritize five populations that bear disproportionately higher HIV burden, one of which is persons who inject drugs (PWID) [1]. PWID accounts for approximately one in ten incident HIV cases [9], with many citing socioeconomic barriers (e.g., homelessness, incarceration) hindering the ability of PWID to access prevention and treatment services for both HIV as well as substance use [10].

Given the populations of people who are vulnerable to HIV due to injection and non-injection use of drugs, outpatient substance use disorder (SUD) treatment centers and OTPs are well-positioned to implement routine HIV testing and diagnose incident cases early in the infection trajectory. In addition, prior research has shown both the feasibility (e.g., improvements in testing rates and receipt of test results compared to off-site referrals) and economic value of on-site HIV testing in SUD treatment programs [11–13]. Yet, despite the need, feasibility and value of on-site HIV testing in these viable settings, most programs do not offer testing, with less than half of U.S. SUD programs and less than one-third of OTPs offering on-site HIV testing [14]. Prior research has noted many significant organizational-level and client-level barriers preventing widespread HIV testing uptake in these treatment settings, including lack of reimbursement and insufficient billing systems, constraints surrounding staffing, resources, training and workflow, and concerns about delivering HIV test results and linkage to care [14–16]. In addition, research has shown greater prioritization and perceived need for hepatitis C virus (HCV) testing compared to HIV testing, given the higher prevalence of HCV compared to HIV within this population [17, 18]. Additionally, the percentage of individuals with chronic HCV infection who are unaware of their infection (approximately 40%) is higher than those with undiagnosed HIV [19]. Despite the availability of better-tolerated, shorter-duration HCV curative treatments, recent CDC data in the U.S. has shown that the number of people with HCV who have initiated treatment has declined over the past few years [19]. Therefore, offering on-site testing services for HCV and HIV has been touted as being more relevant to OTPs than offering on-site testing services for HIV alone. The joint offer of HIV and HCV testing in OTPs [20] is particularly salient, considering that approximately 90% of PWID who seek care in traditional healthcare settings, i.e., non-substance use-related treatment, do not receive any HIV/HCV testing at their clinical visit [21]. As such, more integrated approaches in OTPs may enhance key testing opportunities for high-risk populations to improve the identification of HIV and/or HCV and subsequent active referral for care.

Within this context, the objective of our 3-arm randomized controlled trial (RCT) “*Project I Test: Implementing HIV Testing in Opioid Treatment Programs*” is to focus on addressing commonly cited organizational-level barriers to widespread HIV testing in OTP settings, as well as examine whether the offer of HCV testing in conjunction with HIV testing serves as a motivator for implementation of HIV testing. These goals align with the current NHAS strategy to develop new and expanded implementation of effective, evidence-based, or evidence-informed models for HIV testing that improve convenience and access [1]. The approach we adapted, implemented, and are currently assessing through this RCT utilizes “Practice coaching” (PC), a low-intensity, evidence-based, hands-on approach used to guide implementation of a change initiative, with the change initiative in this study being increased on-site HIV testing in OTPs. PC has been used to implement change in healthcare practices that improve client outcomes, largely through care delivery in primary care settings including increasing preventive service delivery rates, assisting with chronic disease management, and implementing system-level improvements within practice settings [22–26]. The two active PC intervention approaches in this RCT were designed to improve the initial and sustained implementation of on-site HIV testing and linkage to care among OTP clients either alone or in conjunction with HCV testing; rates of HIV testing and linkage to care (as well as their associated cost-effectiveness) of the two PC interventions can then eventually be compared incrementally to one another as well as to an information-only control condition. The purpose of this paper is to discuss these approaches, as well as outline the overall protocol of our Project I Test study, which to our knowledge is the first study to test organizational approaches to increase uptake of HIV and HIV/HCV testing and linkage to care within community-based outpatient programs that provide opioid use disorder (OUD) treatment. Therefore, this study has critical public health implications for understanding how OTP settings can best be supported in the implementation of our innovation of interest (i.e., offering HIV testing on-site and linking PWH to care) and in their sustainment of these improvements, with the ultimate goal of improving HIV-related health outcomes for clients receiving opioid treatment.

## Study objectives

The *primary objective* of the study (Project I Test) is to evaluate the uptake of HIV testing at OTPs, following the implementation of interventions that include practice coach facilitated structured conversations around implementing change, along with provision of relevant resources and documents to support the implementation of (1) HIV testing only, or (2) HIV/HCV testing, and (3) a control condition that provides a package with information only. The *secondary objectives* of the Project I Test study are to evaluate the following: the incremental impact of the HIV/HCV intervention (e.g., proportion of OTP clients tested) on the implementation of HIV testing, compared with the HIV only intervention, and during the initial impact period; the effectiveness of the interventions relative to the control condition, on the sustained impact of HIV testing; and initial impact of HCV testing and sustained impact of HCV testing. The *tertiary objectives* of the Project I Test study are to evaluate the effectiveness of the interventions relative to the control condition on linkage to HIV care among OTP clients who test positive for HIV; linkage to HCV care among OTP clients who test positive for HCV; change in perceived barriers/facilitators to HIV testing; and intervention impact mediated by change in perceived barriers/facilitators. Additional tertiary objectives include evaluating the organizational and environmental characteristics of OTPs that serve as facilitators and barriers to the provision of HIV testing, the sustained implementation of HIV testing, the uptake of testing by OTP clients, and providing timely linkage to care for persons who test positive. The *quaternary objective* is to assess the health outcomes, health care utilization, and cost-effectiveness of the PC interventions compared incrementally to one another and to the control condition. This will allow for assessing the budget required to implement (scale up and sustain) the PC interventions nationally.

## Methods/design

### Study design

**Fig. 1:**
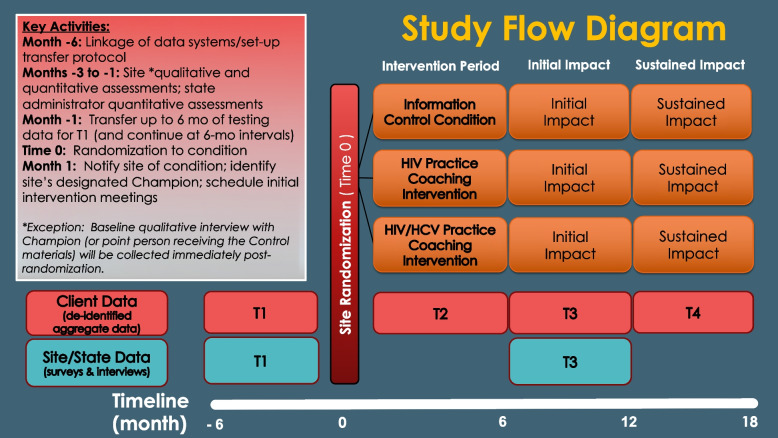
Flow diagram of the trial design The diagram illustrates the progression of sites through the different points of the study

This protocol manuscript follows the SPIRIT reporting guidelines [27]. The design is a 3-arm cluster-RCT of sites treating opioid use disorder in the U.S. Fifty-one OTPs are randomly assigned to one of three conditions (17 sites per condition)—information-only control arm, PC to initiate or increase HIV testing and linkage to care, and PC to initiate or increase HIV and HCV testing and linkage to care (Fig. 1). The study tests the effect of two active evidence-based PC interventions against an informational control on the provision and sustained implementation of on-site HIV testing and linkage to care, and on-site HIV/HCV testing and linkage to care, among OTP clients. The hypotheses in this phase II trial are based on establishing superiority of practice coaching to information only and the HIV + HCV arm to the HIV arm.

### Randomization

Sites are randomized into three groups (HIV PC, HIV/HCV PC, and information-only control condition) in a ratio of 1:1:1 using a blocked randomization scheme to ensure relative balance across time of entry into the study. The allocation sequence for the trial was generated by the study statistician (DF). SAS version 9.4 was used to generate the randomization schedule. The data analyst, who is not involved in the delivery of the intervention, keeps the randomization schedule and sequence secure, and ensures confidentiality and independence of the allocation data. Programs are enrolled in the study before their allocation is revealed. Site personnel are notified to which of the three study conditions the site has been assigned, only after site personnel complete baseline surveys and interviews, and the site is enrolled in the study. Each program is enrolled and informed of their allocation by the data analyst. Due to the nature of the trial, whereby practice coaching content of the interventions are based on allocation, programs and practice coaches will not be blinded to the allocation group. The Principal Investigators and other members of the study team will however remain blinded until primary analyses are completed.

### Eligibility criteria

Site eligibility criteria for this study are as follows: (1) OTP site sees at least 150 unduplicated clients per year; (2) The site is capable and willing to prospectively collect data on the number of clients who (a) are offered any HIV and/or HCV tests; (b) completed these tests; (c) are referred to care/evaluation (and type of referral) if positive); and (d) are linked to care/evaluation within 30 days of diagnosis of HIV and/or HCV; (3) The site is capable and willing to provide aggregate client testing data within demographic categories of gender and race/ethnicity and data on HIV/HCV test reimbursement processes and outcomes; (4) the site is able to select staff willing to consent to participate in study surveys, qualitative interviews, and intervention coaching throughout the study. Sites in which over 50% of clients served in the prior 6 months were HIV or HCV tested are excluded. To be eligible to participate in the study’s site surveys, interviews, and intervention activities, individuals must be site personnel employed within one of the 51 enrolled sites.

### Study settings and recruitment

The sampling frame consists of all opioid treatment programs/sites in the 2017 National Survey of Substance Abuse Treatment Service (N-SSATS), a national census of all US substance use treatment facilities, that have a minimum client census of 150 clients per year. The study draws on a random sampling of 500 eligible sites from this sampling frame and will draw additional sites as needed. A total of 51 eligible sites will be enrolled in the trial. Recruitment occurs through email and telephone contact. Site leadership (e.g., Chief Executive Officer, Director) are contacted, informed about the study and invited to complete a screening process to determine the OTP’s eligibility to participate in the study. If interested in participating in the study, the site leader completes a brief screening by telephone interview (after providing verbal consent) or via self-administered survey to determine the site’s eligibility to participate. Enrollment consists of obtaining a signed form letter from each participating site, outlining the various study activities in which the site personnel will participate. The site leader must also complete an acknowledgment from noting that participation in the study is voluntary and that there will be no impact to any individual employee of the site for not participating. Participants in this study consist of the professional staff working at eligible treatment programs/sites around the country that treat clients with opioid use disorder. Staff at selected sites that accept the invitation to participate are interviewed and complete brief surveys to confirm that they meet eligibility criteria. We also recruit, via email, directors working at state substance use authorities. We will conduct a survey of state policies and guidelines relevant to HIV and HCV testing. To participate in state surveys, individuals must be directors at state substance use authorities in the participating sites’ states.

Sites complete all surveys and related evaluations according to the study timeline. Participants may retract their consent to participate in the study and may do so at any time before or during the study. Once a site or staff member participating in the study withdraws from the study during treatment, their data is not excluded from our analysis, but will only be included for the time period in which they provided data.

A number of procedures are in place to promote retention in the study for the duration of the planned intervention. The primary strategies to improve retention in the interventions in this trial are twofold. The first is our incentive structure. Participating sites receive monetary incentives during their 2-year involvement. The payments are given once after completing their initial data collection plan (typically within 2 weeks of randomization) and a second/final time after the site completes the second of four aggregate data transfers. Personnel questionnaires and interviews are compensated at $40 and $50 (respectively); per each site’s discretion, these incentives are either issued directly to the personnel completing them or pooled into a single site-wide incentive (e.g., staff luncheon). This is intended to prevent participants from providing partially completed questionnaires, not adhering to treatment as delivered, or withdrawing from the study after enrollment. Secondly, the PCs work with the sites to encourage them to participate and adhere to the intervention sessions/timeline/window. PCs are mindful and respectful of the sites’ time and busy schedules and therefore ensure that they meet their scheduling needs. The collaborative nature of the intervention helps as the PC will assist the site in setting goals/action items and help brainstorm and discover ways to implement change. Intervention adherence is part building relationships and part the site staff’s time and motivation. Contacting the site to encourage them and move them along is part of the success.

### Conceptual/theoretical framework

The Consolidated Framework for Implementation Research (CFIR) framework was the basis for identifying essential factors supporting or impeding the adoption of testing [28]. The five CFIR domains we considered in developing the PC interventions are based on contexts that influence the implementation, effectiveness, and sustainability of our approach: inner setting (e.g., networks, climate, readiness), outer setting (e.g., client needs and resources, peer pressure, incentives), intervention characteristics (e.g., evidence strength, adaptability, cost), individual characteristics (e.g., self-efficacy, knowledge, beliefs), and the implementation process (e.g., planning, engaging, executing, evaluating). The implementation of the PC interventions was then guided by the stage theory of organizational change. Change theories guide the implementation of interventions, as well as the evaluation [29–31]. Stage theory posits that organizations move through four sequential stages as they change or adopt an innovation: awareness, adoption, implementation, and institutionalization (see Table 1). Each stage involves specific strategies that are matched to that stage, the particular OTP, and factors external to the organization (e.g., how CDC guidelines are implemented in the particular OTP’s state). We provide details of the specific steps to be taken within each of the 4 sequential stages of the interventions below. PC is tailored to the context of the OTP, focusing on organizational change.

**Table 1:** Stages of Organizational Change: Project I-Test^a^

Concept		Definition	Application
1. Define problem (Awareness Stage)	1. Sense unsatisfied demands on a system 2. Search for possible responses 3. Evaluate alternatives 4. Decide to adopt course of action	Problems recognized and analyzed; solutions sought and evaluated	Involve management and other personnel in awareness-raising activities
2. Initiate Action (Adoption Stage)	5. Initiate action within system	Policy or directive formulated; resources for beginning change allocated	Provide process consultation to inform decision makers and implementers about what is involved in adoption
3. Implementation Stage	6. Implement the change	Innovation implemented; reactions occur and role changes occur	Provide training, technical, and problem-solving assistance
4. Institutionalization Stage	7. Institutionalize the change	Policy or program becomes entrenched in organization; new goals and values internalized	Identify high-level Champion (someone with decision making power or influence, beyond the implementation Champion), work to overcome obstacles to institutionalization, and create structures for integration

^a^Excerpted from Glanz, K., & Bishop, D. B. (2010) [32]

## Study interventions

The two PC interventions are manualized and training of Practice Coaches (PCs) emphasizes the importance of adhering to the manual that corresponds to a site’s assigned intervention condition (i.e., preventing drift). To ensure consistency of intervention delivery across all PCs, the PCs co-facilitated the first few intervention sessions. PCs also co-facilitate some intervention sessions later in the study to ensure that they are still delivering the intervention in the same manner and adhering to the manuals. Additionally, the Intervention Director conducts regularly scheduled “peer to peer” conference calls to discuss difficulties and successes in conducting the PC interventions; to facilitate the PCs learning from and supporting each other; and to facilitate receiving support and feedback from the Intervention Director.

All participants are provided with information and resources, per their intervention allocation. Programs are discouraged from additional treatments that are not according to the study protocol, during the intervention period. Participants will be required to report all treatments that are not according to the treatment protocol, i.e., an initiative that supports the adoption of HIV or HCV testing delivered by a coach.

### Practice coaching

Skilled PCs serve as a resource for programs. PC’s work includes helping the site leader to identify an organizational change agent/champion, who will lead the program’s on-site testing effort and serve as the primary liaison to the study team. The Champion is supported by a Change Team, who are key staff identified by the Champion, with guidance from the PC, i.e., individuals with high-level of commitment to organizational change and improving testing practices. PC activities will encompass (1) pre-implementation assessment, feedback and goal setting, (2) information on the provision of HIV or HIV/HCV testing and linkage to care, (3) leveraging existing resources (e.g., staff, space, equipment) to improve the HIV or the HIV/HCV service delivery system and facilitate billing and reimbursement for testing, (4) technical and decision support for reimbursement of testing services, and (5) improved linkages to medical care and city, state, and federal sources for testing resources. PCs support sites by helping them navigate resources, as well as support the site in addressing potential barriers, including, but not limited to, human resources, staff training, and resource allocation. PCs engage OTPs over 6 months to guide them through the process of improving the initial and sustained implementation of HIV or HIV/HCV testing services and linkage to care (see Fig. 1).

The treatments in this study are two active interventions: PC for HIV testing, and PC for HIV/HCV testing.

- *HIV PC condition*
  In the HIV PC intervention, the PCs work with the programs to (a) establish capabilities, reimbursement systems and/or partnerships necessary to support HIV testing and evidence-based linkage to care and (b) reduce barriers (e.g., staffing, training) to the initial and sustained provision of on-site HIV testing. The intervention occurs over 6 months (approximately 29 weeks) and consists of four distinct phases, each involving evidence-based stages designed to establish competency in the implementation of organizational change towards establishing (or increasing) HIV testing among OTP clientele.
- *HIV and hepatitis C virus (HIV/HCV) PC condition*
  The HIV/HCV PC intervention leverages the HIV PC intervention and follows the same sequence of steps. However, in this intervention, PCs work with the sites to establish practices for both HIV and HCV testing.

### Linkage to HIV and/or HCV medical care within both PC conditions

Sites in both PC intervention conditions are coached to link clients who receive an HIV-positive test result (either antibody or RNA) to follow-up medical care within 30 days of diagnosis. Coaching includes familiarization of approaches to linkage to HIV care (i.e., evidence-based Anti-Retroviral Treatment and Access to Services (ARTAS) counseling). PCs also support sites by helping them navigate resources, focus their use of linkage to care materials, as well as support the site in addressing potential barriers, including, but not limited to, human resources, staff training for linkage, and resource allocation to facilitate linkage to care services. Sites assigned to the HIV/HCV PC intervention condition also receive coaching preventive self-care and protecting liver function from further harm through reducing or eliminating alcohol consumption, and Hepatitis A and B vaccination, as appropriate. PCs also link clients who receive an HCV-positive test result (either antibody or RNA) to follow-up evaluation and/or medical care within 30 days of HCV diagnosis.

## Control condition

### Provision of information

The administrators and/or designated personnel within the OTPs assigned to the information control condition receive the official National Institute on Drug Abuse (NIDA)/Substance Abuse and Mental Services Administration (SAMHSA) Blending Initiative product, “HIV Rapid Testing in Substance Abuse Treatment Programs,” that we will provide to OTPs to educate and motivate them about the importance of offering on-site HIV testing. They will also receive an electronic link and/or hard copy of the ARTAS implementation manual and training information as well as information about Pre-Exposure Prophylaxis (PrEP), a daily medication that serves as an HIV prevention tool for individuals who are HIV-negative but at substantial risk of acquiring HIV infection. Resources generated from the HIV rapid testing blending initiative product include a fact sheet, resource guide, marketing materials, and an Excel-based budgeting tool. In addition to the HIV-specific materials, the Website provides opportunities for training, self-study progress, workshops, and distance learning.

## Description of intervention stages

*Awareness Phase 1* is concerned with raising interest and generating support for the intervention with senior management by defining the problem (i.e., local HIV prevalence, resource allocation for HIV testing), and identifying possible solutions such as establishing a billing and reimbursement system for HIV testing services, training and motivating staff to test clients for HIV, and connecting with a health care center so that procedures are in place to link clients who test HIV-positive to care.

Phase 1 includes five steps: Step 1 is a teleconference call between the PC and the site’s Leader, including advice to select a champion, with appropriate interest, knowledge base, skill set, and leadership capacity. Step 2 is a teleconference call between the PC and the site’s designated champion. Step 3 involves the PC’s comprehensive assessment of barriers and facilitators to the provision, client uptake, and reimbursement of HIV testing services. This assessment is based on a structured interview conducted by the PC. Step 4 is a concentrated in-person or virtual workshop and with the champion and key staff from the site. PCs review the goals and objectives of practice coaching, knowledge-based HIV information, the provision of HIV testing services, quality improvement, monitoring and evaluation tools, billing and reimbursement for HIV testing (including alternatives such as securing free test kits from the local health department and/or establishing Memoranda of Understanding / Agreement (MOU/ MOAs) with the health department and/or other community-based organizations to provide HIV testing services within the site), introduction to evidence-based linkage to care strategies as well as a review of roles/responsibilities and data capture forms. One purpose of the workshop is for the PC to synthesize results of the site’s comprehensive barriers/facilitators assessment and pre-intervention performance data and present these results to the site’s champion(s) and key staff, providing constructive feedback on identified barriers and potential solutions. Another key purpose of the visit is creating an action plan that is tailored to the OTP’s context and culture and that addresses identifying/securing resources needed to initiate or increase on-site testing. Step 5 is a debrief phone call with site Champion and Change Team to review and discuss the action plan for testing. This interaction with the PC also presents opportunities for sites to ask additional questions.

*Adoption Phase 2* begins when an organization decides to commit to and initiate an innovation or evidence-based intervention (e.g., on-site testing); this phase includes refining the action plan for on-site testing. The champion and key staff (the “change team”) use the plan-do-study-act (PDSA) method, a structure for iteratively guiding goal setting and planning. PCs assist change teams and provide tools to facilitate relationship building with stakeholders for adopting and implementing system/OTP-wide changes, specific strategies to achieve HIV testing goals through appropriate mechanisms. Specific intervention activities in this stage include (1) ongoing video or traditional teleconference call meetings utilizing the PDSA format. Additionally, (2) PCs will guide the champions and the OTP change team in engaging organizational “gatekeepers” to build consensus and negotiate any needed action plan modifications without jeopardizing the integrity of the stated goals. (3) PCs will meet (by phone or video conference) with the change teams biweekly, and as needed, regularly to support any necessary iterations between steps 1 and 2.

*Implementation Phase 3* is the process of integrating an innovation within a setting, involving identification of (and changes to) practice patterns or organizational structures as necessary to overcome identified barriers. This involves the technical aspects of providing HIV testing, including staff training and procurement of materials as well as the support needed for the introduction of change. For linkage to HIV care, it is critical to identify the facilities and teams to which people are linked for follow-up care, and engagement of new sets of stakeholders may be required. Additionally, building staff capacity and motivation for testing and linkage to care is crucial for sustained implementation. PCs provide support on the following: (1) optimizing workflow (e.g., what type of HIV testing to implement, when to provide testing), (2) application of CDC and state-level HIV testing and linkage to care guidelines, (3) development and maintenance of a training and quality assurance program to ensure front-line staff have initial and continued knowledge, support, and motivation to provide HIV testing/linkage to care, (4) assistance with the effective use of billing and reimbursements systems (established in Phase 2) for sites with the capacity to bill (e.g., processes to facilitate coding of services, timely submission of claim), and initiation of efforts to translate information and resources for setting-up infrastructure for billing among sites that are not already billing for services, (5) support tools to help sites engage clients (e.g., testing campaigns) and promote the uptake of HIV testing, and (6) increasing utilization of community resources that enhance the site’s capacity to provide HIV services.

Each site is given access to self-management tools as well as national and state resource guides accessible via study-managed folders in Box.com, which include online links to organizations such as the CDC, Health Resources and Services Administration (HRSA) and SAMHSA, the site’s state health department, and a repository of guidelines and updated information on HIV testing and linkage to care practices. Sites are also provided with support tools, such as flowcharts and spreadsheets to track clients across the HIV care continuum. Additionally, sites have the opportunity to share other state and national resources pertinent to testing and linkage to care with each other (if they wish) by posting these resources to a shared space in Box.com. As appropriate, PCs serve as liaisons, connecting staff at each site with resources in their community to support testing and linkage to care for clients who test positive. PCs meet with program teams regularly (via video conference or telephone) to support the tailoring and implementation of their action plan and system-wide changes to achieve their stated goals. While PCs guide and support the initiation, sustained implementation, and measuring of changes to HIV testing practices, PCs do not lead the actual implementation of the proposed changes.

To facilitate inter-organizational learning during the Implementation Phase, PCs consider ways to connect sites willing to share their learning experiences with their OTP peers. Conference calls between sites within the same intervention condition are encouraged and arranged by PCs when sites are willing to participate in this activity. The calls allow participating programs to learn about various implementation strategies and seek guidance from colleagues on strategies to overcome different barriers. The calls also serve as a uniquely informative place for sites to learn about “late breaking,” on-the-ground changes in policies affecting services, funding, and organization, and what may (or may not) be relevant from one region to another. Attending sites set the agenda for (and facilitate) the interactive calls (not the PC). However, the PC may attend the call and provide input at the sites’ request.

*Institutionalization Phase 4* refers to the capacity of OTPs to maintain the integration of the innovation into routine practice and achieve the expected coverage of the intervention (i.e., increase in the proportion of clients’ HIV testing) over an extended period of time. At this stage, top managers and stakeholders are of great importance to continued investments in resources and training and establishing processes for monitoring/evaluation. These activities are necessary for sustaining improvements.

Substantial organizational change literature shows that once adopted and successfully implemented, practices or innovations are often maintained over time without the need for continuing intervention. The sustainability of organizational-level changes is often associated with changes in organizational practices rather than the behavior of individuals. Changes to organizational practices may, however, have a direct beneficial impact on individual behavior. Additionally, interventions are considered sustainable when implementation strategies are maintained, and relevant activities (i.e., as described in Phases 1–3) and resources are allocated in-line with stated goals. Therefore, PCs will focus on five main activities to enable sustainability:

1. Establish a process for continuous monitoring and evaluation of organizational change and outcomes, including uptake of testing.
2. Facilitate planning of a course of action for adapting to changes in funding that occur over time and identifying new funding streams for testing.
3. Support the continued benefits to clients (uptake of HIV testing and linkage to follow-up care for persons who test positive) by assisting sites to implement key activities and allocate resources, both financial and human, accordingly.
4. Assist sites to develop a plan for institutionalizing the services provided by the PCs (i.e., lessons learned from the PC, with the champion serving as an inter-organizational coach).
5. Develop a plan for continued engagement of organizational stakeholders and generating client interest in HIV testing, receiving test results, and engaging in medical care.

## Study assessments

Three types of data are collected, *client data, site data*, and *state data*. The assessments used in the study consist of three quantitative surveys with treatment program staff (i.e., treatment program administrators, treatment program clinical staff), and state administrators; and qualitative interviews with treatment program directors and study champions (see Table 2). The treatment program administrator survey measures structure and service setting, client characteristics, staffing characteristics, program guidelines, barriers to care, and perceptions. The treatment program clinician survey measures training, knowledge, experience, barriers, and perceptions. The state administrator survey covers policies/regulations, reimbursement, and prioritization of testing services. The qualitative interviews address in-depth discussion about testing services offered at the site, barriers and facilitators to offering HIV/HCV testing services and linkage to care, attitudes towards services and training at the site, and organizational readiness for change.

**Table 2:** Duration of study and assessment/activities schedule Once a given site enrolls in the study, its duration of participation is approximately 24 months broken into four distinct 6-month-long intervals, as visually depicted in the Fig. 1 study flow diagram. Because the date on which a site is randomized to one of the three study conditions is considered to be Time = “0”, the timeline for a given site is depicted as running from month − 6 to month 18. The various assessmentss and activities occur within this timeline as shown

Assessment	T1 (months − 6 to − 1)	T2 (months 1 to 6)	T3 (months 7 to 12)	T4 (months 13 to 18)
Aggregate (de-identified) Client Data Summary	X	X	X	X
Site Administrator Survey	X		X	
Clinician Survey	X		X	
State Administrator Survey	X		X	
Qualitative Interview—Site Administrator/ Leader	X		X	
^a^Qualitative Interview—Champion or Point Person	X		X	
^b^Brief Demographic Questionnaire		X		
^b^Readiness for Change Questionnaire		X		
^b^Practice Coaching Intervention Acceptability Questionnaire		X		
^c^Practice Coach Interaction Form		X		
^c^Quarterly Peer-to-Peer Evaluation		X		
^b^Cost Survey		X		
^b^Cost Interview (as needed)		X		

^a^The baseline Qualitative Interview for the Champion (or point person receiving the information control materials) will be conducted immediately post-randomization so intervention sites have time to identify who will be the Champion

^b^Practice Coaches and Site personnel within sites assigned to an intervention condition will complete these assessments

^c^Practice Coaches will complete these activities/assessments throughout the intervention period to help inform cost analyses and (if one or both interventions are successful) the development of a refined manual to be used for “real-world” PC implementation

The intervention/control period is approximately 29 weeks or 6 months in duration. Interventionists (Practice Coaches) will engage OTPs over the 6-month intervention to guide them through the process of improving the provision and sustained implementation of HIV or HIV/HCV testing services and linkage to care. Approximately 16 sessions (including an on-site visit) will occur during the intervention; the number of sessions will be greater in the first intervention phase and taper toward the last phase

## Outcomes

### Primary outcome

The primary outcome analysis will compare the PC interventions with the control condition on the initial impact of HIV testing. Using HIV testing data provided by programs, the outcome is measured by the mean proportion of OTP clients tested during the period 7–12 months after randomization (“initial impact”, T3), while controlling for HIV testing during the baseline period (T1).

### Secondary outcomes

The secondary outcome analysis will examine the incremental impact of the HIV/HCV testing intervention condition (i.e., difference in HIV testing rates for the HIV/HCV condition) compared with the HIV testing condition, on the proportion of OTP clients tested for HIV during the period 7–12 months after randomization (T3). Other secondary outcome analyses will examine the impact of the PC interventions on the sustained impact of HIV testing (proportion of OTP clients tested during T4, 12–18 months after randomization), compared with T3, initial impact of HCV testing (proportion of OTP clients tested during T3), and sustained impact of HCV testing (proportion of OTP clients tested during T4, compared with T3).

### Tertiary outcome measures

The effectiveness of the interventions relative to the control condition will be examined for tertiary outcomes: linkage to HIV care among OTP clients who tested positive for HIV, linkage to HCV care among OTP clients who tested positive for HCV, and change in perceived barriers/facilitators to HIV testing. We will also examine, using mixed methods, the interventions’ impact mediated by changes in perceived barriers/facilitators; the impact of the PC interventions on OTPs’ organizational and environmental characteristics that serve as facilitators and barriers to the initial and sustained implementation of HIV testing, the uptake of testing by OTP clients, and providing timely linkage to care for persons who test positive. While the intervention emphasizes on-site testing, study outcomes may assess any testing, either on- or off-site, to measure potential spillover effects of the intervention.

### Quaternary outcome measures

We will determine health outcomes, health care utilization, and cost-effectiveness of the PC interventions, and compare them incrementally to one another and to the control condition. We will also assess the budget required to implement (scale up and sustain) the PC interventions nationally.

## Data sources

We use various approaches to collect data to measure outcomes and covariates (see Table 3). Study sites, upon enrollment in the study, are provided with a spreadsheet which they may use to assist in compiling aggregate de-identified data summaries, including HIV/HCV testing data. These data are transferred from sites at 6-month intervals and are checked for consistency. We use REDCap Survey data capture tools, with automatic range and consistency checks for quantitative survey data collection. PCs track the intervention process and record these data in structured forms, i.e., Practice Coach Interaction Form (PCIF). Some of the intervention process data are collected and managed using REDCap, and other intervention process data are collected using electronic collection forms. Qualitative interview data, including audio recordings and transcriptions, are collected via digital audio recorders. All data are stored securely on an encrypted and password-protected server. All personal data of participants, both program and staff, are assigned a unique identifier that is stored on a secure server available only to data analysts and researchers with approved access to the database. Data analysis will only include non-identifiable data. Prior to analyses of the site surveys, factor structure and reliability of scales will be documented and all variables will be assessed for appropriate statistical distributions for analysis. Any missing data will be accommodated using multiple imputation.

**Table 3:**
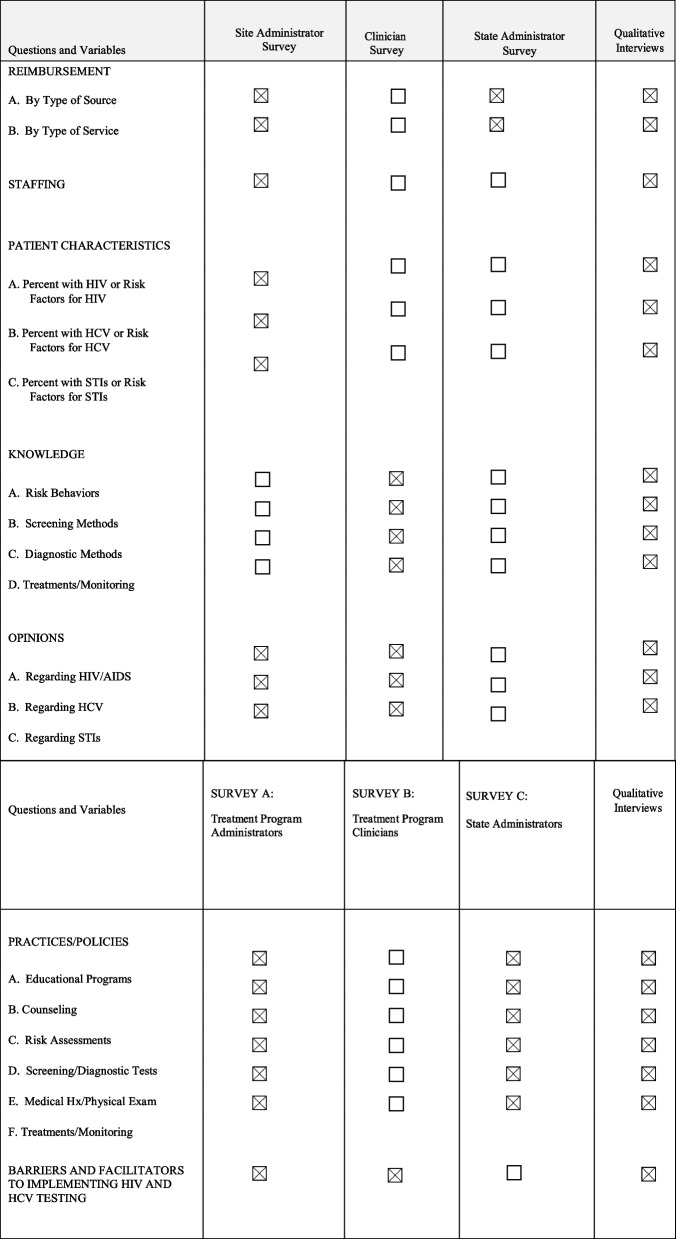
Measures and Approaches to Data Collection

## Data monitoring

To ensure monitoring of other study-related participant safety events or incidents, procedures regarding confidentiality and data integrity are continually monitored and regularly audited. Members of the PI team meet regularly (e.g., biweekly) during the study period to review trial progress.

Developed and implemented by the PIs, who constitute the data safety and monitoring board (DSMB), the data and safety monitoring plan (DSMP) assures minimal risk and data integrity in this study. The plan assures that all data collection procedures concur with all local, state, and federal guidelines. To assure integrity of data and safety, all aspects of the program are monitored, including informed consent procedures, data collection and quality (i.e., review for statistical anomalies), fidelity of the practice coaching intervention to the intervention manual, and adherence of the qualitative interviews to the interview guide. The data monitoring center, as well as DSMB, examines accumulating data to assure protection of participants’ safety while the study’s scientific goals are being met. The DSMB conducts periodic reviews of accumulating safety and effectiveness data and determines whether there is support for continuation of the study, or evidence that study procedures should be changed, or if the study should be halted for reasons relating to the safety of the study participants, the effectiveness of the treatment under study, or inadequate study progress. Because the study supports the implementation of HIV testing based on a practice coaching intervention, adverse events related to the intervention are not expected. However, unintended adverse events may include unauthorized disclosure of confidential information and potential discomfort/embarrassment related to answering quantitative and/or qualitative questions concerning clinical and organizational practices, perceptions, and attitudes about HIV/HCV testing; and potential violations of confidentiality. In the event that an adverse event or otherwise untoward incident occurs as a direct result of or in the context of the project, we closely follow IRB directives and reporting policies. Specifically, we report to the appropriate IRBs within 10 working days, in writing, all serious adverse or otherwise untoward events associated with procedures. To ensure monitoring of other study-related participant safety events or incidents, procedures regarding confidentiality and data integrity are continually monitored and regularly audited. The PIs promptly inform other Co-Is and NIDA staff of any proposed changes in site enrollment, intervention implementation, or in the protocol that are relevant to safety, as well as any actions taken by the IRBs as a result of their continuing review of the project. In the event of any major changes in the status of an ongoing protocol (which occurs only with IRB approval), the PIs inform NIDA’s program officer and the DSMB immediately. Such changes would include, but are not limited to protocol amendments, temporary suspension of site initiation/commencement, changes in informed consent or IRB approval status, termination of participation by the site and/or site personnel, or other problems or issues that could affect the human subjects in the study.

## Power and sample size

Statistical power and sample size were determined using a simulation programmed in SAS 9.3. The simulation generated data with a range of intra-class correlations (ICC) from 0.04 to 0.08, and an information control condition with a proportion of clients’ HIV testing of 20% as found in the control condition in CTN0032, a study assessing the relative effectiveness of three HIV testing strategies on increasing receipt of test results and reducing HIV risk behaviors among patients seen at drug use treatment centers [13]. A sample size of 51 OTPs and an average of about 100 clients per site per 6-month period provides over 95% power for the primary outcome and 85% power for the secondary outcome if the proportion of clients’ HIV testing in the HIV PC condition is 30% (absolute difference of 10% from control condition) and the proportion of clients’ HIV testing in the HIV/HCV PC condition is 41% (absolute difference of 11% from the HIV PC condition) for all expected levels of ICC (0.04 to 0.08). Should some sites drop out, there is still over 95% power for the primary and 83% power for the secondary, as long as 45 sites remain in the study. For the quasi-experimental evaluation of the blending product, the study will have over 80% power to uncover an absolute change in proportion testing for HIV of 6 to 7%. For analysis of change in proportion of facilitators/barriers, the study will have over 80% power to uncover a significant difference in change if the difference in change is 0.5 to 0.65 of a standard deviation, a medium effect size.

## Empirical analysis

The primary outcome analysis will test the hypothesis that the two PC interventions will result in significantly higher proportions of clients tested for HIV than the control condition during the “initial impact” period (7–12 months post-randomization or T3), controlling for the proportion of clients tested during the baseline period (T1). We will use a generalized estimating equation (GEE) model with a binary distribution and logit link. The model will include four 6-month periods: T1 (months − 6 to − 1)—prior to randomization, T2 (months 1–6)—during intervention/control period, T3 (months 7–12)—initial impact, and T4 (months 13–18)—sustained impact. Time and participants are both nested within site. However, time is not nested within participants in the primary analysis. Individuals within a site may be more alike (correlated) than are individuals between sites, which will be accounted for in the GEE by inclusion of the working correlation matrix within site and the sandwich estimator for standard errors. The model will include gender and race/ethnicity, and geographic region as control variables. The primary tests of H1 will be done using a contrast of testing differences across conditions in the proportion of clients tested during T3, controlling for the proportion of clients tested pre-randomization (T1). All hypotheses will use the intention-to-treat approach and include all available data for the sites that are randomized.

The secondary and tertiary outcome analyses will use similar GEE methods as described for the primary outcome measure. The secondary outcome will test the hypothesis that the HIV/HCV PC intervention will result in significantly higher proportions of clients tested for HIV than the HIV PC intervention during the initial impact period (7–12 months post-randomization or T3), controlling for the proportion of clients tested during the baseline period (T1). Of note, the design of the primary outcome and the secondary outcome comparing the HIV/HCV intervention to the HIV intervention are an orthogonal decomposition of the 2-degree of freedom test of whether the 3 interventions are significantly different from each other. As such, there is still a type I error of 0.05 for these two hypotheses jointly and there is no reason for error correction for using 2 tests to determine differences among the interventions.

Other secondary measures will examine, for example, the impact of the PC interventions on the provision and sustainability of HIV testing (T4), the impact of the PC interventions on initial impact of HCV testing (T3). The tertiary outcome analysis related to linkage to care will evaluate the effectiveness of the interventions relative to the control condition on linkage to HIV care among OTP clients who tested positive for HIV, or for HCV, as well as change in perceived barriers/facilitators to HIV testing.

We will use mixed methods to evaluate the impact of the PC interventions and the OTPs’ organizational and environmental characteristics that serve as facilitators and barriers to the provision and uptake of HIV testing (T3), sustained implementation of HIV testing (T4), and improving timely linkage to care for persons who test positive. We will use a multilevel GEE model to examine whether change in perceived barrier/facilitators mediates intervention impact on HIV testing (T4). Mediation will be assessed by the product of coefficients method.

## Cost analysis

The quaternary outcome includes determining the costs associated with health outcomes and evaluating the cost-effectiveness of the interventions. We plan to complete a cost-effectiveness analysis, using data from the I Test trial to populate a simulation model, the Hepatitis C Cost-Effectiveness model (HEP-CE). HEP-CE is a microsimulation of HCV disease progression, screening, and treatment. The cost-effectiveness analysis will assume a health sector cost perspective and a lifetime horizon. We will test the hypothesis that the incremental cost-effectiveness ratio (ICER) for the HIV PC intervention will be below a commonly cited US willingness-to-pay threshold (< $100,000/quality-adjusted life years (QALY)) and therefore more economically attractive than the control condition. That is I Test will be cost-effective assuming a societal willingness to pay of $100,000 per QALY gained. Our other hypothesis is that the ICER for the HIV/HCV PC intervention will be more economically attractive than the HIV PC intervention. The study will follow a proven model of effective collaboration among the intervention team and computer simulation modelers to evaluate the health outcomes, health care utilization, and cost-effectiveness of the PC interventions [11]. We will use I Test data to inform key model parameters, such as rates of linkage to care with and without the I Test intervention, as well as the cost of the intervention. The established micro-costing techniques will be used to identify the costs of delivering the PC interventions, including personnel and non-personnel costs incurred centrally to deliver the intervention and incurred at the OTPs to participate in the intervention and conduct follow-up activities (excluding time required for research activities). Model outputs include cases identified, linked to care, and treated, as well as life expectancy, quality-adjusted life expectancy, costs, and cost-effectiveness.

The micro-costing results and data on the characteristics of clients at each of the OTPs will be used as inputs to analyses conducted using the HEP-CE microsimulation model of HIV and HCV infections [33, 34]. These analyses will evaluate the incremental health outcomes, healthcare utilization, and cost-effectiveness of the PC interventions, considering the lifetime benefits and costs of linking to treatment clients newly identified as HIV-infected and as HCV-infected. The HEP-CE model will be used to conduct sensitivity analyses that consider a range of assumptions about key model parameters such as prevalence of undiagnosed HIV and HCV infection, effectiveness of linkage to care, likelihood of treatment initiation once linked, and likelihood of screening and linkage in the absence of the intervention. Separately, micro-costing data will be used to explore the budgetary requirements to scale up the PC interventions nationally, including the budget implications for participating OTPs. Sensitivity analyses will consider different scenarios for the sustainability of the interventions depending on level of success at institutionalizing testing practices.

## Qualitative coding and data analysis

The development and application of a multilevel coding scheme is an integral component of the data analysis process. At the highest level of the coding hierarchy are the primary analytic foci, coded as headings. Specific dimensions of the headings are assigned core codes, while dimensions of the core codes are assigned sub-codes. We will use ATLAS.ti, a software program for qualitative analysis, to facilitate the analysis. Seven steps will be used to develop the coding scheme: (1) identify the principal issues discussed by participants; (2) construct definitions of the primary analytic themes; (3) develop and apply core codes (themes) and sub-codes (sub-themes) to the initial set of interviews; (4) develop a provisional coding scheme; (5) test the coding scheme by applying it to a subsample (*n* = 15) of interviews, (6) refine the provisional coding scheme; (7) have two research team members independently apply the coding scheme to a new subsample (*n* = 15) of interviews; (8) have them meet to reconcile differences in their application of the codes; (9) Refine the coding scheme as needed and finalize it; and (10) apply the finalized coding scheme to the full data set. Inter-coder reliability will be assessed with kappa statistic.

After all transcripts have been coded, the study team will extract and examine the content of text linked to specific core codes and sub-codes and identify ways in which certain themes are analytically related. Identified relationships among themes may lead to more refined data searches. Once patterns of relationships among themes and issues are established, the study team will try to identify participants’ accounts that support or refute these patterns. Identifying and accounting for cases that “deviate” from an interpretative pattern enables us to test and confirm the pattern’s validity and robustness*.* Finally, the study team will attempt to map themes onto the relevant domains of the CFIR framework to assess the framework’s adequacy in identifying all the important factors supporting or impeding the adoption of testing. If emergent in these analyses, it will be possible to identify pathways through which adoption (of lack of adoption) of testing evolves in the PC versus the control conditions.

## Discussion

Our PC interventions, if shown to be effective and cost-effective, could be used at multiple levels to provide ongoing support to OTPs in delivering HIV/HCV testing. This promising approach should be adaptable to address HIV testing in other settings, including pharmacies, dental care settings, and community centers. To our knowledge, this study is the first to test organizational approaches to increase HIV and HIV/HCV testing strategies in OTPs. If successful, SAMHSA, HRSA, the AIDS Education and Training Center, the Addiction Technology Transfer Centers and other community-based agencies at the national, state, and local levels could use our organizational support approaches to provide ongoing support to SUD treatment programs in delivering HIV and HCV testing. This proposal is also well-aligned with the new National Institutes of Health (NIH)-wide guidelines for priorities for HIV/AIDS grants. The first priority is to reduce the incidence of HIV/AIDS and one of the main goals is to develop, test, and implement strategies to improve HIV testing and entry into prevention services.

Despite evidence highlighting the effectiveness and economic value in on-site HIV testing in SUD treatment programs, current testing practices are inadequate. There is an overall need for expanded HIV testing among persons who use substances, particularly in underutilized settings where high-risk persons receive health services. The I Test project is one of the first comprehensive studies to develop and test a PC intervention to support the adoption and implementation of HIV and HIV testing in opioid treatment programs. It is also novel in that it employs a study design that accounts for the integration of HIV and HCV testing in treatment programs, with a focus on linkage to care [35]. Additionally, the translation of findings from this study is central and is supported by the cost analysis. In light of the Affordable Care Act (ACA) facilitating initiatives to increase the provision and sustainability of HIV testing, therein lies a pivotal opportunity for OTP treatment sites to increase their continuous implementation of HIV testing and timely linkage to care. With cost barriers being largely negated, organizational barriers remain the predominant limiting factor in OTP sites’ uptake of testing; as such, our study is among the first to systematically test implementation strategies at the organizational level to promote the delivery of HIV testing in OTPs. By introducing a PC approach shown to be effective in primary care settings into OTP sites, our study aims to help sites navigate their reimbursement systems and mitigate staff-related barriers with the ultimate goal of bolstering timely HIV testing and linkage to care for those most in need.

## Trial status

The trial is in the data collection stage, with the recruitment and randomization process nearly completed. Recruitment began 6/14/2017 and is expected to continue until late 2023. The protocol version number is 7.0, with date of 4/7/2021.

### Supplementary Information

**Additional file 1. **A completed SPIRIT checklist.

**Additional file 2. **WHO Trial Registration Data Set.

## Data Availability

Following the publication of the trial results, anonymized datasets arising from this trial will be exclusively accessible, for a period of time, to researchers from the I Test study. All data can however be made available upon reasonable request. Authorship eligibility for any publication from the trial in this protocol will adhere to the International Committee of Medical Journal Editor (ICMJE) guidelines, as well as internal processes and procedures.

## Abbreviations

ARTAS: Anti-Retroviral Treatment and Access to Services
CDC: Centers for Disease Control
CFIR: Consolidated Framework for Implementation Research
CUMC: Columbia University Medical Center
DSMB: Data Safety Monitoring Board
DSMP: Data Safety Monitoring Plan
GEE: Generalized estimating equation
HCV: Hepatitis C virus
HRSA: Health Resources and Services Administration
ICC: Intra-class correlations
ICER: Incremental cost-effectiveness ratio
IRB: Institutional Review Board
MOU/MOAs: Memoranda of Understanding / Agreement
NIH: National Institutes of Health
NHAS: National HIV/AIDS Strategy
NIDA: National Institute on Drug Abuse
OTP: Opioid treatment programs
OUD: Opioid use disorder
PC: Practice coaching
PDSA: Plan-do-study-act
PrEP: Pre-exposure prophylaxis
PWH: People with HIV
PWID: Persons who inject drugs
QALY: Quality-adjusted life years
RCT: Randomized controlled trial
SAMHSA: Substance Abuse and Mental Health Services Administration
SUD: Substance use disorder
U.S.: United States
USPSTF: US Preventive Services Task Force
WHO: World Health Organization
