# Supplementary material for: A study protocol for Project I-Test: a cluster randomized controlled trial of a practice coaching intervention to increase HIV testing in substance use treatment programs
Source: Trials. 2023 Sep 26;24:609. doi: 10.1186/s13063-023-07602-8 (PMC10521543; doi:10.1186/s13063-023-07602-8)
Supplement: Supplementary file 2 — Additional file 2. WHO Trial Registration Data Set. [file 13063_2023_7602_MOESM2_ESM.docx]

| **Data category** | **Information** |
| --- | --- |
| Primary registry and trial identifying number | ClinicalTrials.gov |
|  | NCT03135886 |
| Date of registration in primary registry | 2-May-17 |
| Secondary identifying numbers | AAAQ9986 (Columbia IRB protocol number) R01DA043130 (U.S. NIH Grant/Contract) |
| Source(s) of monetary or material support | National Institute on Drug Abuse (NIDA) |
| Primary sponsor | National Institute on Drug Abuse (NIDA) |
| Secondary sponsor(s) | None |
| Contact for public queries | Jemima A. Frimpong, PhD; New York University Abu Dhabi, PO BOX 129188, Saadiyat Island, Abu Dhabi, UAE; jafrimpong@nyu.edu |
| Contact for scientific queries | Jemima A. Frimpong, PhD; New York University Abu Dhabi, PO BOX 129188, Saadiyat Island, Abu Dhabi, UAE; jafrimpong@nyu.edu |
| Public title | *Project iTest* |
| Scientific title | *Project I Test: Implementing HIV Testing in Opioid Treatment Programs* |
| Countries of recruitment | United States |
| Health condition(s) or problem(s) studied | 1) HIV testing and linkage to care and 2) HIV/Hepatitis C virus (HCV) testing and linkage to care among patients seeking/receiving substance use disorder treatment |
| Intervention(s) | Experimental: HIV Testing Practice Coaching Intervention Group The HIV Testing Practice Coaching (PC) Intervention is designed to improve the provision and sustained implementation of on-site HIV testing and linkage to care among OTP patients. |
|  | Experimental: HIV and HCV Testing Practice Coaching Intervention Group The HIV and HCV Testing Practice Coaching (PC) Intervention will leverage the HIV PC intervention and follow the same interventional steps described above, and, in addition, provide information and training to support joint HIV/HCV testing and linkage to care among OTP patients. |
|  | Information Control Group: The administrators of OTPs assigned to the control condition will receive a website link to and hard copy of the NIDA/SAMHSA Blending Initiative product for HIV rapid testing. |
| Key inclusion and exclusion criteria | Ages eligible for study: ≥18 years (adult, older adult) |
|  | Sexes eligible for study: All |
|  | Accepts healthy volunteers: Yes |
|  | Inclusion criteria:  Eligible sites must: - See at least 150 unduplicated patients/year/site - Be capable and willing to prospectively collect data on the number of patients who a) are offered any HIV and/or HCV tests; b) completed these tests; c) are referred to care/evaluation (and type of referral) if positive; and d) are linked to care/evaluation within 30 days of diagnosis - Be capable and willing to provide patient demographics, testing data within demographic categories of gender and race/ethnicity (in aggregate) and data on HIV/HCV test reimbursement processes and outcomes - Have key staff willing to consent to participate in study surveys, qualitative interviews and intervention coaching throughout the study |
|  | Exclusion criteria:  Sites will be excluded if: - Over 50% of patients served in the prior 6 months were HIV or HCV tested - They are terminated via PI decision/discretion |
| Study type | Interventional |
|  | Allocation: randomized |
|  | Intervention model: parallel assignment |
|  | Masking: None (open label) |
|  | Primary purpose: Health Services Research |
|  | Phase II |
| Date of first enrolment | Jun-17 |
| Target sample size | 51 OTP sites |
| Recruitment status | Recruiting |
| Primary outcome(s) | Proportion of OTP patients HIV tested at 6 months post intervention or control, while controlling for HIV testing during the baseline period (T1) [ Time Frame: The period 7-12 months post site-randomization (T3). ]  The primary outcome measure will be a contrast of the proportion of OTP patients HIV tested during T3, controlling for HIV testing at baseline (T1). The primary test will be whether the proportion of patients tested across the two PC interventions--HIV Testing PC Intervention Group and HIV/HCV Testing PC Intervention Group--differs from the proportion of patients tested in the Information Control Group. |
| Key secondary outcomes | Proportion of OTP patients HIV tested at 12 months post intervention or control [ Time Frame: The period 13-18 months post site-randomization. ]  The secondary outcome measure will be the proportion of OTP patients HIV tested during T4, controlling for HIV testing at baseline (T1). One test will be whether the proportion of patients tested across the two PC interventions--HIV Testing PC Intervention Group and HIV/HCV Testing PC Intervention Group--differs from the proportion of patients tested in the Information Control Group. A second test will be whether the proportion of OTP patients HIV tested during T4 in the HIV Testing PC Intervention Group differs from the proportion of patients tested in the HIV/HCV Testing PC Intervention Group. |
